# Utilization of an automated machine learning approach for the detection of granular corneal dystrophy via slit lamp photographs

**DOI:** 10.1186/s12886-025-04324-0

**Published:** 2025-11-22

**Authors:** Negin Yavari, S. Saeed Mohammadi, Jared T. Sokol, Dalia El Feky, MohammadBagher Rajabi, Jia-Horung Hung, Christopher Or, Osama Elaraby, Frances A. Anover, Aim-On Saengsirinavin, Amir Akhavanrezayat, Azadeh Mobasserian, Ngoc Trong Tuong Than, Jingli Guo, Yue Bai, Cigdem Yasar, Natalie A. Afshari, Charles C. Lin, Quan Dong Nguyen

**Affiliations:** 1 Byers Eye Institute, Stanford University, Palo Alto, CA USA; 2 Department of Ophthalmology, Feinberg School of Medicine, Northwestern University, Chicago, IL USA; 3 Department of Ophthalmology, Tanta University, Tanta, Egypt; 4 Shiley Eye Institute, University of San Diego, La Jolla, CA USA; 5 Spencer Center for Vision Research, Byers Eye Institute, Stanford University School of Medicine, 2370 Watson Court, Suite 200, Palo Alto, CA USA

**Keywords:** Machine learning, Automated machine learning, Granular corneal dystrophy

## Abstract

**Introduction:**

This study aims to apply automated machine learning (AutoML) techniques for the diagnosis of granular corneal dystrophy (GCD), a rare inherited condition characterized by progressive protein deposition in the corneal stroma.

**Methods:**

Patients diagnosed with GCD who had slit-lamp photographs of the affected eye(s) were enrolled in the study. Individuals with concomitant corneal conditions, ungradable imaging data, or uncertain diagnoses were excluded from the study. Slit-lamp photos depicting the GCD and non-GCD were obtained from the Byers Eye Institute, Stanford University. Image processing included resizing and cropping, focusing solely on the cornea. A deep learning model was subsequently deployed, utilizing Vertex-AI, the AutoML platform developed by Google (Menlo Park, CA). The area under the precision‒recall curve (AUPRC) was plotted, and the sensitivity, specificity, positive predictive value (PPV), accuracy (AC), and F1 score were calculated.

**Results:**

The model was trained on a dataset comprising 223 images, consisting of 72 GCD and 151 non-GCD images. One hundred seventy six images were used for training, 24 were used for validation, and 23 were used for testing the model. The AUPRC for the model was 0.995 and precision and recall were both 95.70% at a confidence threshold of 0.5. The sensitivity, specificity, PPV, AC, and F1 score of the model were 93.30%, 100%, 100%, 95.70%, and 0.965, respectively.

**Conclusions:**

A clinician-derived AutoML model successfully identified GCD from slit lamp photographs with high accuracy.

## Introduction

Corneal dystrophies are rare hereditary conditions that affect the cornea, typically presenting as bilateral, symmetrical, and slowly progressive, and may occur independently of systemic factors [1]. They are characterized by abnormal deposits within the corneal layers and are categorized into epithelial/subepithelial, epithelial-stromal, stromal, and endothelial dystrophies [2–4].

Granular corneal dystrophy (GCD) is an autosomal dominant disease caused by a point mutation in the transforming growth factor-β (TGFB) gene, although it can also arise sporadically due to de novo mutations or mosaicism [1, 5]. GCD typically involves the central corneal stroma, leaving a clear zone between the affected area and the limbus. Earlier in the disease, there are faint subepithelial opacities [6]. As the disease progresses, whitish, snowflakes or breadcrumb-like opacities are present throughout the corneal stroma. Classically, the epithelium remains intact, leading to a good visual prognosis, and interventions such as corneal grafting are rarely needed [7]. However, some individuals may experience significant impairment, necessitating therapeutic interventions such as phototherapeutic keratectomy [6]. Recurrence of opacities post-keratectomy may also require additional procedures [6, 7].

Slit-lamp examination and photography are established diagnostic methods for corneal diseases, providing opportunities for both qualitative and quantitative analysis of corneal deposits [2]. While confocal microscopy and anterior segment optical coherence tomography (AS-OCT) can be used to help diagnose this condition, a definitive diagnostic tool for accurately detecting GCD is currently lacking. Genetic testing can also identify mutations in the TGFB gene; however, it may not be widely available or affordable in some regions [8]. Several studies have demonstrated the utility of artificial intelligence (AI) in the field of ophthalmology [9, 10]. AI systems represent a novel approach for analyzing and identifying patterns within extensive datasets, transforming them into valuable tools for assisting in clinical decision-making. Machine learning (ML) is a subset of AI in which computer-generated algorithms and models can make predictions on the basis of data. Automated ML (AutoML) simplifies the process by removing the need for technical coding skills. Studies on AutoML, including ours, involve creating a model that can autonomously select appropriate algorithms and handle data preprocessing tasks to achieve accurate and efficient predictions with no programming expertise [11]. Thus, this study aims to utilize AutoML for the detection of GCD from slit lamp images.

## Methods

The study received approval from the Institutional Review Board (IRB) of Stanford University. The research was conducted in accordance with the Declaration of Helsinki, the United States Code of Federal Regulations Title 21, and the Harmonized Tripartite Guidelines for Good Clinical Practice (1996). Owing to the retrospective design of the study, the IRB waived the requirement for written informed consent.

### Dataset

**Fig. 1:**
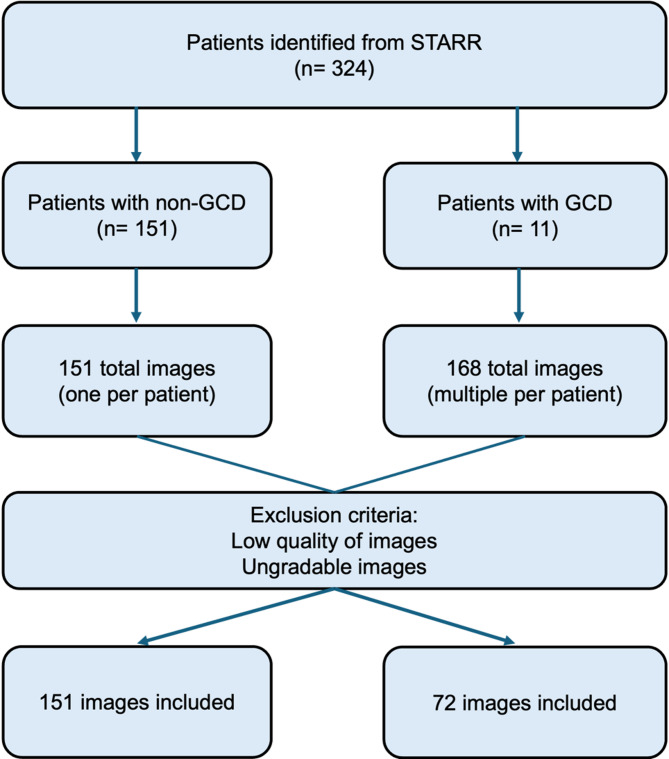
Flow diagram of the study sample

To evaluate the effectiveness of AutoML in diagnosing GCD, a dataset comprising both corneas with and without GCD was used (Fig. 1). To compile this dataset, patients diagnosed with GCD were identified via the Stanford Research Repository (STARR) tools. A total of 168 slit lamp photos of the affected patients (*n* = 11) with GCD were subsequently extracted and deidentified. Among the 168 GCD images, 72 were of high quality and could be used in the model. For the control group, images of unique patients without GCD who were seen by cornea specialists were obtained and deidentified. Given the availability of 72 high-quality images representing GCD, we estimated the appropriate number of control (non-GCD) images to maintain class balance and optimize model performance in Vertex AI. A case-to-control ratio of approximately 1:2 was chosen, resulting in 151 control images. This ratio was selected to ensure adequate representation of the minority class (GCD) while reducing bias during model training. Maintaining at least 30% representation of the minority class has been shown to improve the stability of key performance metrics, including precision, recall, and F1 score, particularly in small datasets. This strategy aligns with recommended practices for building robust machine learning models under data-limited conditions. The control dataset included images from patients diagnosed with conditions such as Fuchs’ corneal dystrophy, corneal ulcer/scar, Fuchs’ epithelial dystrophy, cataract, corneal edema, keratitis, pterygium, and healthy cornea. This selection allowed us to better reflect real-world conditions and assess the model’s ability to differentiate between cases and controls. All 223 images in the dataset represented unique patients to ensure data independence and maintain patient level splits across the training, validation, and testing sets.

### Image acquisition

All images were captured using a HaagStreit IM 900 imaging system (Camera Module CM03, HaagStreit, Koeniz, Switzerland) equipped with a digital imaging system. Images were acquired in a darkened room with the participant seated and fixating in primary gaze position. A consistent working distance and magnification were maintained across all sessions. We used diffuse illumination with a beam width of 10 mm and height of 8 mm. The angle between the illumination and observation systems was set to 30–45 degrees to optimize visualization of stromal changes. The light intensity was kept constant to avoid overexposure or washout. No filters were applied during image acquisition. Additionally, all images were reviewed for adequate focus, illumination, and contrast. Images were included if they demonstrated clear visualization of the cornea without motion blur, overexposure, shadows, or obstructing artifacts. Images failing to meet these criteria were excluded to ensure consistency across both GCD and control groups (Fig. 1).

### Image processing

The extracted slit lamp photos were anonymized and stored as deidentified DICOM files on the Stanford Nero Secured Computing Platform. The images were resized to dimensions of 128 × 128 × 64 pixels to ensure consistency in all images. Prior to uploading to Google Vertex AI, all images were converted to JPEG format. Manual contrast enhancement or histogram equalization was not applied. No custom augmentation was applied locally, as Vertex AI’s AutoML image classification model automatically applies internal data augmentation during training, which may include random flipping, rotation, and brightness/contrast adjustment depending on platform optimization routines.

### Model training

**Fig. 2:**
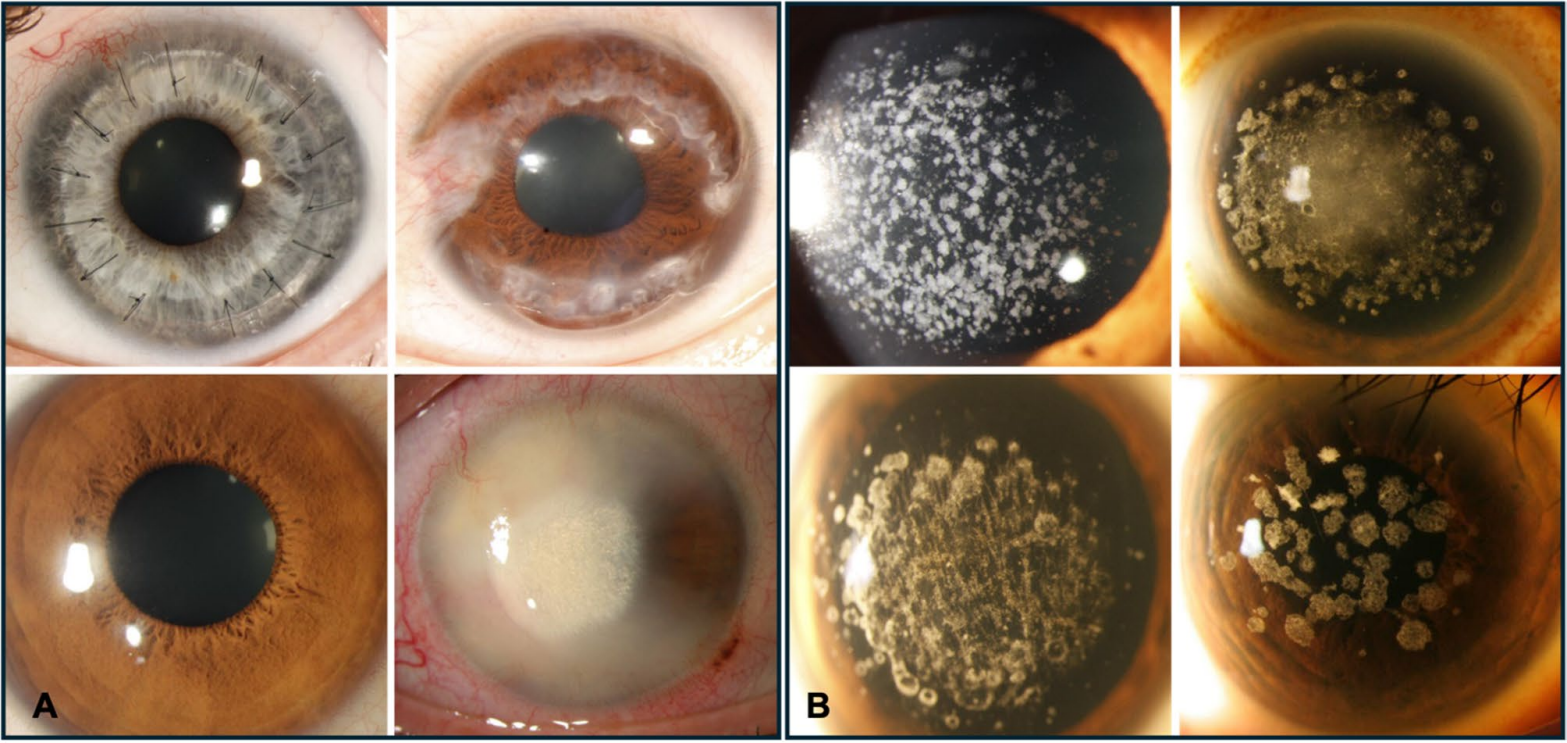
Images of (**A**) Non-granular corneal dystrophies and (**B**) Granular corneal dystrophies diagnosed by a cornea specialist

A dataset using single-label classifications was established for the training of the model. Image analysis was carried out in the “us-central” region of the Google Cloud AutoML platform. Each image was labeled as either ‘non-granular corneal dystrophy’ or ‘granular corneal dystrophy’, in accordance with diagnoses provided by physicians (Fig. 2).

This dataset was segmented into three subsets using a random number generator: 78.9% for training, 10.8% for validation, and 10.3% for testing. Model hyperparameters (e.g., learning rate, number of layers, batch size, and optimizer selection) were tuned automatically by the platform. No manual hyperparameter adjustments were made. 25.7 node hours were used for model training.

While the exact neural network architecture used is proprietary and not disclosed in full by Google, Vertex AI commonly relies on transfer learning from base models such as EfficientNet or ResNet. The platform selects and optimizes the base model architecture internally during training. Consequently, we were not able to specify or modify the underlying model architecture directly.

### Performance metrics

The AutoML platform autonomously produces metrics to gauge the effectiveness of the model it has trained. It computes the area under the precision‒recall curve (AUPRC) as a measure of the model’s proficiency in accurately categorizing images. Moreover, the platform generates confusion matrices that expose the counts of true positives, true negatives, false positives, and false negatives. These counts are essential for deriving key performance metrics, including sensitivity, specificity, positive predictive value (PPV), overall accuracy (AC), and the F1 score.

### Explainability and saliency mapping configuration

To evaluate the interpretability of the deep learning model, we utilized the Vertex Explainable AI within the Vertex AI platform. Two saliency-based visualization techniques, Integrated Gradients (IG) and eXtended Region Activation Mapping (XRAI), were applied. These methods were used to generate heatmaps over the input images, revealing regions that contributed to the prediction, such as granular opacities, when predicting GCD. For both methods, the default settings provided by Vertex AI were used. IG used a baseline (reference) image consisting of all-black pixels, and XRAI used internal segmentation of the image to guide attribution region computation. Attribution maps were overlaid on original images and qualitatively assessed for localization. No additional tuning or custom settings were applied to the saliency mapping.

## Results

In total, 223 images, consisting of 72 GCD cases and 151 controls, were included in the training and self-evaluation of AutoML. One hundred seventy six images were used for training, 24 images were used for validation, and 23 images were used for testing the model.

**Fig. 3:**
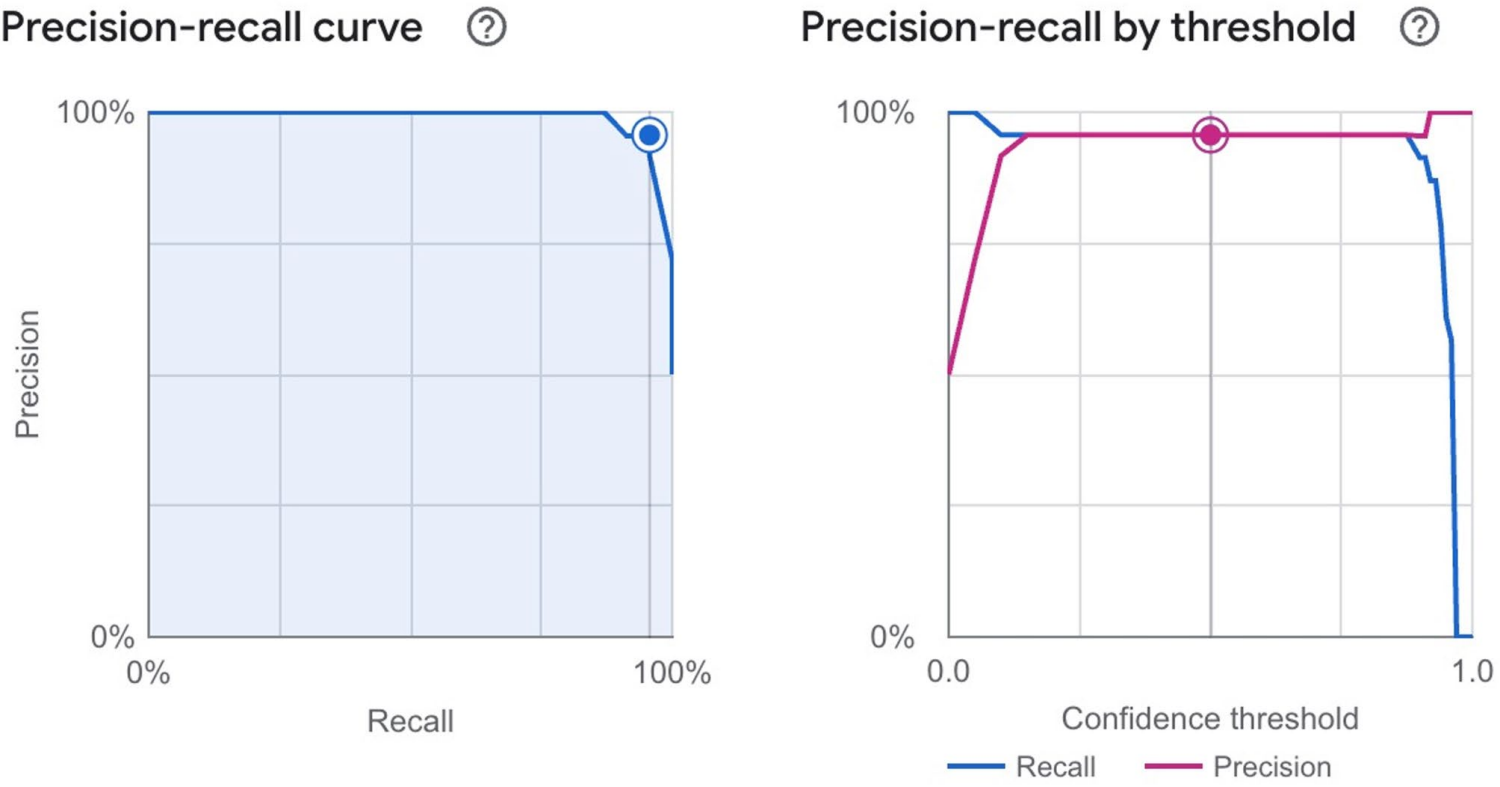
**A** Precision‒recall curve and **B** Precision‒recall by confidence threshold for granular corneal dystrophy prediction model from slit-lamp photographs

The model had an AUPRC of 0.995 (Fig. 3). Moreover, the precision and recall at confidence threshold of 0.5 were 95.70% and 95.70%, respectively (Fig. 3).

**Fig. 4:**
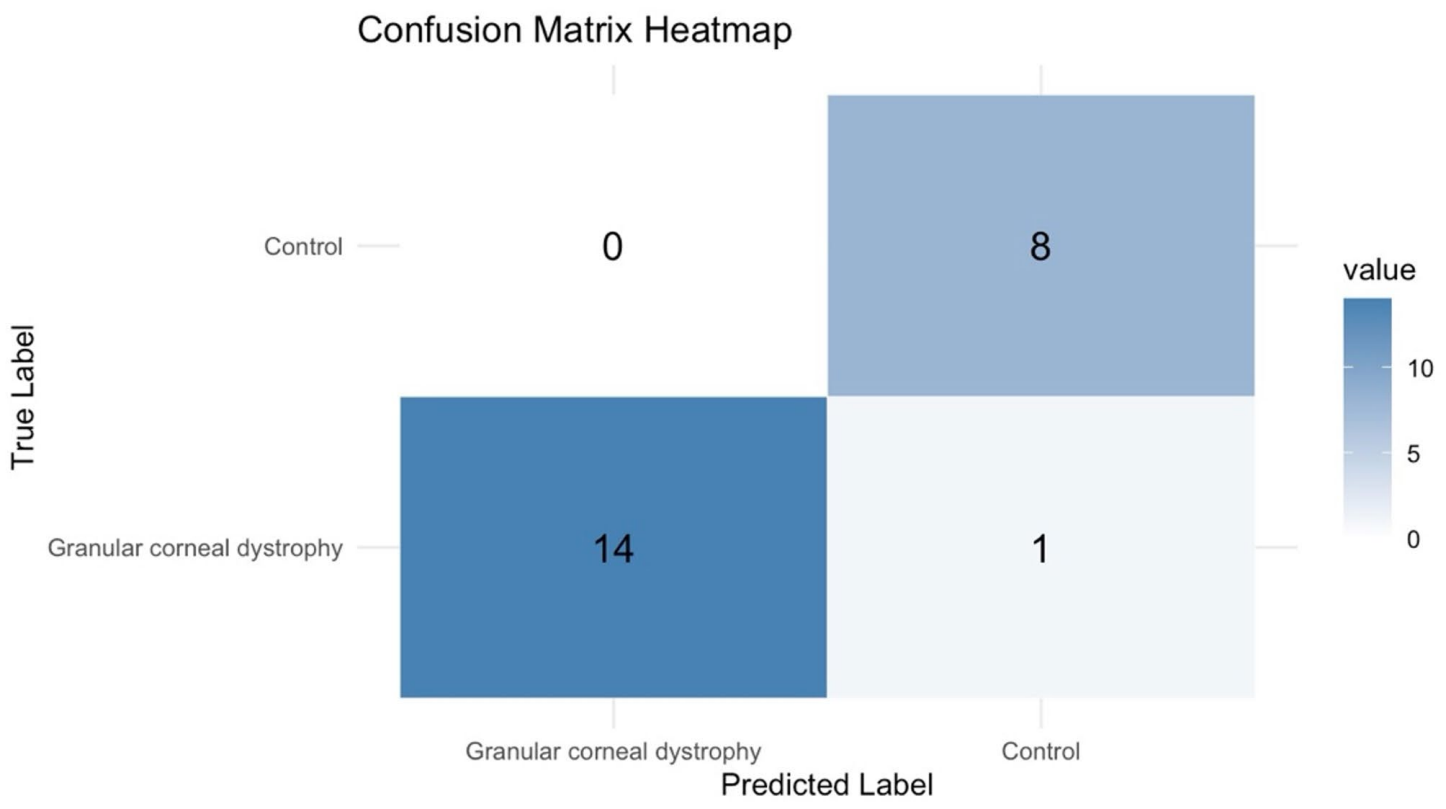
Confusion matrix of the trained model

The confusion matrix generated from the testing set revealed 14 true positives, zero false positives, eight true negatives, and one false negative. Evaluation metrics calculated from the matrix showed 93.3% sensitivity, 100% specificity, 100% PPV, 95.7% AC, and 0.965 F1 score (Fig. 4).

**Fig. 5:**
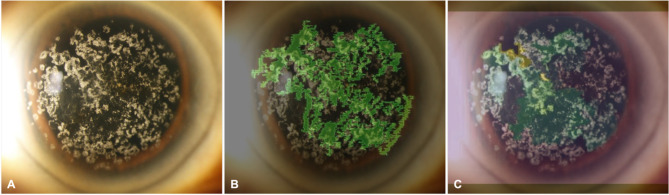
Model explainability visualization. **A** Original image of granular corneal dystrophy. **B** Integrated Gradients heatmap. **C** XRAI heatmap. Green and yellow overlays indicate regions contributing most to the model’s prediction

As shown in Fig. 5, image A displays the original slit-lamp image of a cornea with clinically confirmed GCD. Image B presents the attribution heatmap generated using IG, highlighting in green the regions that contributed most to the model’s prediction. These regions broadly overlap with the granular opacities characteristic of GCD. Image C shows the XRAI heatmap, which segments the image into regions and attributes saliency scores, with green and yellow overlays denoting high-importance zones. XRAI produced more localized attribution patterns, emphasizing denser granular clusters. Both techniques demonstrated that the model’s predictions were driven by clinically relevant features corresponding to known GCD pathology.

## Discussion

This study evaluated the diagnostic accuracy of a code-free, accessible AI model built via AutoML. The field of ML has evolved with the introduction of AutoML. Traditional ML requires a deep understanding of data preprocessing, feature engineering, model selection, and tuning, often demanding significant time and expertise. However, AutoML automates many of these tasks, enabling users with limited ML expertise to develop competitive models more efficiently and allowing for rapid prototyping and deployment [12]. Our model was developed via AutoML and was tested internally on slit lamp images of patients with and without GCD at a tertiary eye center.

Despite the availability of numerous diagnostic tools, there is currently no definitive diagnostic tool that allows both ophthalmologists and non-ophthalmologists to accurately differentiate GCD from other types of corneal diseases. In addition, the effectiveness of existing tools heavily depends on the expertise of cornea specialists in conducting examinations, making diagnoses, and interpreting corneal images. Ophthalmology, an outpatient-focused specialty with frequent use of various imaging techniques, is well suited for integrating AI to enhance patient care. The abundance of imaging and patient data available in this field can be leveraged to develop AI algorithms, particularly for cornea-related diseases [13–15].

The diagnosis and management of corneal diseases often rely on various imaging modalities, such as slit-lamp photography, AS-OCT, and corneal topography [9, 10]. These modalities could be utilized in training AI algorithms to assist in automatically diagnosing corneal and anterior segment conditions such as posterior capsule opacification [16], dry eye syndrome [14], keratoconus [13], and Fuchs endothelial dystrophy [15].

With the abundance of data available, ML enables the development of models that can make accurate clinical predictions. Even though healthcare researchers are familiar with clinical data, they still often lack the traditional ML expertise necessary to apply these techniques to big data sources and therefore rely on expert data scientists to develop predictive models. Even with the input from data scientists, datasets must be prepared for ML via manual feature engineering techniques, which is not trivial as the performance of a given ML algorithm relies heavily on the quality of the input features [17].

By incorporating AI to increase the efficacy of traditional ML and alleviate its challenges, automated feature engineering has emerged [12]. AutoML automates image processing steps, including cleaning, transforming, and normalizing data, to identify suitable algorithms. Using AutoML offers several advantages that help to enhance the ML process. It eliminates the need for manual trial and error, which saves a significant amount of time in model building and optimization. It requires less expertise in ML, making it accessible to a wider range of users and enabling the handling of large datasets and complex ML tasks. Moreover, it enables the creation of more accurate models and reduces bias in ML models by automating feature engineering and model selection processes [12].

Several publications have explored the use of AutoML for medical image classification and diagnosis in other ocular diseases, including diabetic retinopathy [18, 19], trachoma [20], graft failure post-keratoplasty [21], pachychoroid disease [22], posterior capsule opacification and proliferative vitreoretinopathy [16, 23]. All of these studies demonstrated the accuracy and feasibility of AutoML models for identifying ocular diseases, which are consistent with our findings. Our study is the first to evaluate the application of AutoML to identify GCD. Our results demonstrate the feasibility of using AutoML to detect GCD from silt-lamp photographs. The image classification model differentiating slit-lamp photographs of patients with and without GCD had excellent performance with an AUPRC of 0.995, precision and recall of 95.70% (confidence threshold of 0.5), sensitivity of 93.3%, specificity of 100%, PPV of 100%, AC of 95.7%, and F1 score of 0.965.

Limitations of this study include the small size of the dataset, which reflects the overall rarity of GCD. Our dataset also comprises a larger number of images with one abnormal condition, which might overfit the results. Additionally, future studies with other corneal dystrophies and larger sample sizes would help to validate these results. In addition, collaboration with other institutions to increase the number of images of patients with GCD could enhance dataset diversity and ensure the model performs robustly across different devices, settings, and populations. Although this study demonstrates the feasibility of using an AutoML platform to classify GCD from clinical slit-lamp images, we acknowledge that the limited number of high-quality test images presents a constraint on the generalizability of our findings. Notably, Google Vertex AI AutoML uses transfer learning as part of its model development pipeline, leveraging pretrained convolutional neural networks and fine-tuning them to the task-specific dataset. In future work, additional strategies such as advanced data augmentation, incorporation of external datasets for validation, and exploration of few-shot learning techniques may help further improve model robustness in small-data environments, particularly for rare diseases such as GCD. These approaches can address the current limitations and support the development of more generalizable AI models in ophthalmology.

## Conclusion

AutoML is a viable technology with high accuracy in detecting GCD from slit-lamp photographs without the need for specialized programming skills, allowing ophthalmologists and non-ophthalmologists to leverage ML directly without relying on data scientists or engineers.

## Data Availability

Data will be available to the corresponding author upon reasonable request.

## Abbreviations

AC: Accuracy
AI: Artificial intelligence
AS-OCT: Anterior segment optical coherence tomography
AUPRC: Area under the precision‒recall curve
AutoML: Automated machine learning
GCD: Granular corneal dystrophy
IRB: Institutional review board
ML: Machine learning
PPV: Positive predictive value
STARR: Stanford Research Repository
TGFB: Transforming growth factor-β
XRAI: eXtended Region Activation Mapping
IG: Integrated Gradients
